# Microbiota Changes in Fathers Consuming a High Prebiotic Fiber Diet Have Minimal Effects on Male and Female Offspring in Rats

**DOI:** 10.3390/nu13030820

**Published:** 2021-03-02

**Authors:** Faye Chleilat, Alana Schick, Raylene A. Reimer

**Affiliations:** 1 Faculty of Kinesiology, University of Calgary, Calgary, AB T2N 1N4, Canada; fatima.chleilat1@ucalgary.ca; 2 International Microbiome Centre, Cumming School of Medicine, University of Calgary, Calgary, AB T2N 4N1, Canada; alana.schick@bcchr.ca; 3 Department of Biochemistry & Molecular Biology, Cumming School of Medicine, University of Calgary, Calgary, AB T2N 4N1, Canada

**Keywords:** oligofructose, gut microbiota, short-chain fatty acids, paternal programming, triglyceride

## Abstract

Background: Consuming a diet high in prebiotic fiber has been associated with improved metabolic and gut microbial parameters intergenerationally, although studies have been limited to maternal intake with no studies examining this effect in a paternal model. Method: Male Sprague Dawley rats were allocated to either (1) control or (2) oligofructose-supplemented diet for nine weeks and then mated. Offspring consumed control diet until 16 weeks of age. Bodyweight, body composition, glycemia, hepatic triglycerides, gastrointestinal hormones, and gut microbiota composition were measured in fathers and offspring. Results: Paternal energy intake was reduced, while satiety inducing peptide tyrosine tyrosine (PYY) gut hormone was increased in prebiotic versus control fathers. Increased serum PYY persisted in female prebiotic adult offspring. Hepatic triglycerides were decreased in prebiotic fathers with a similar trend (*p* = 0.07) seen in female offspring. Gut microbial composition showed significantly reduced alpha diversity in prebiotic fathers at 9 and 12 weeks of age (*p* < 0.001), as well as concurrent differences in beta diversity (*p* < 0.001), characterized by differences in Bifidobacteriaceae, Lactobacillaceae and Erysipelotrichaceae, and particularly *Bifidobacterium animalis*. Female prebiotic offspring had higher alpha diversity at 3 and 9 weeks of age (*p* < 0.002) and differences in beta diversity at 15 weeks of age (*p* = 0.04). Increases in *Bacteroidetes* in female offspring and Christensenellaceae in male offspring were seen at nine weeks of age. Conclusions: Although paternal prebiotic intake before conception improves metabolic and microbiota outcomes in fathers, effects on offspring were limited with increased serum satiety hormone levels and changes to only select gut bacteria.

## 1. Introduction

Substantial evidence shows that maternal, fetal, and neonatal microbiota elicit transient and long-lasting impacts on health that is based on the presence of microbes themselves as well as the metabolites they produce [1,2,3]. Prebiotics, defined as a substrate that is selectively utilized by host microorganisms conferring a health benefit [4], have been used to beneficially modulate the gut microbiota in animal and human studies. For example, prebiotic supplementation and particularly oligofructose (OFS) in rodents during gestation and lactation has resulted in improvements in bodyweight, body composition and colon length in offspring [5], reductions in immune-related incidents [6], and improved glucose tolerance, insulin sensitivity, and hepatic steatosis in offspring [7]. Importantly, maternal intake of prebiotic oligofructose has been shown to selectively alter obese maternal gut microbiota composition and significantly enhance the abundance of the health-promoting genera *Bifidobacterium* [8]. Maternal prebiotic oligofructose intake resulted in increased satiety hormone levels and a serum metabolomics signature that suggested prebiotic supplementation of a maternal high fat/sucrose diet could reduce the insulin resistance of obese pregnant rats with benefits for their offspring [8].

Whether or not a paternal diet high in prebiotics could similarly benefit offspring health is not known but there is mounting evidence for the impact of paternal environment, including metabolism, physiology, body composition, and diet on sperm quality, fetal development, and offspring health into adulthood [9]. In fact, growing animal and human research from epidemiological studies have deduced that the period before conception is vital in influencing the development of health of prospective generations [9]. Mirroring female reproductive fitness, male reproductive health been associated with multiple environmental factors, including nutrition [9]. Paternal high fat diet consumption for 10 weeks before conception was shown to affect pancreatic β-cell function, and impair insulin secretion and glucose homeostasis in fathers and offspring, although the detrimental effects could be decreased if offspring consumed a control diet [10]. Furthermore, paternal low protein diet in a mouse model perturbed the expression of genes modulating hepatic lipid and cholesterol biosynthesis in offspring [11]. Still, while our understanding of the impact of paternal diet on offspring health is increasing, there remains much to be investigated. To our knowledge, no studies have examined whether paternal prebiotic supplementation with oligofructose, before conception impacts offspring health. Our objective was to examine if a paternal prebiotic-rich diet during pre-conception affects the microbial and metabolic status of the fathers and their offspring.

## 2. Materials and Methods

### 2.1. Animal Model and Dietary Treatment

Twenty-four male Sprague Dawley rats (Charles River Laboratories, Montreal, QC, Canada) were housed in a temperature and humidity-controlled facility with a 12-h light/dark cycle. One day following arrival, animals were randomized to 1 of 2 nutritionally complete experimental diets from the age of 3 to 12 weeks: (1) control AIN-93G diet (from 3 to 9 weeks of age) and AIN-93M (from 10 to 12 weeks of age) (Dyets Inc., Bethlehem, PA, USA) or (2) 10% (wt/wt) prebiotic. The 10% prebiotic diet was prepared in house by mixing 900 g of AIN-93G (from 3 to 9 weeks of age) or AIN-93M (from 10 to 12 weeks of age) with 100 g of oligofructose (Orafti P95, Beneo-Orafti, Mannheim, Germany). OFS was selected as the prebiotic because it has been shown in various human and animal studies to improve anthropometric outcomes, satiety, and health promoting bacteria [4,7,12,13]. Experimental diet composition can be found in Table 1. At 12 of age, fathers were co-housed with a virgin female Sprague Dawley rat during the active dark cycle, with AIN-93G diet given ad libitum. Once a copulation plug was identified, dams were moved to a single-housed cage throughout their pregnancy and during lactation. Dams were given a control, AIN-93G diet and water ad libitum. Within 24 h of birth, litters were culled to *n* = 5 females and *n* = 5 males. Cross-fostering with litters from the same dietary intervention group took place if litters were less than *n* = 10. Male and female offspring from the same litter were designated *n* = 1. Due to their young age during the study (from 3 weeks of age), fathers and offspring were co-housed with an age-matched rat in the same treatment group except when breeding took place. Offspring from 3 to 16 weeks of age consumed a control diet (AIN-93G and/or AIN-93M). Food intake was measured every 3 weeks, for 3 consecutive days each time. This study was approved by the University of Calgary Animal Care Committee (AC18-0074) and conformed to the Guide to the Care and Use of Laboratory Animals.

### 2.2. Body Weight and Composition

Fathers and offspring were weighed weekly. One day before euthanasia, body composition was determined using a dual energy X-ray absorptiometry (DXA) scan and software for small animals (Hologic ODR 4500, Hologic, Bedford, MA, USA). Animals were lightly anaesthetized using isoflurane to ensure stillness. Recorded measurements included: bone mineral content/density (BMC/BMD) (g and g/cm^2^), fat mass (g), lean mass (g), and body fat %.

### 2.3. Oral Glucose and Insulin Tolerance Tests

Following an overnight fast, fathers (at 10 weeks of age) and offspring (at 14 weeks of age) underwent an oral glucose tolerance test (OGTT). OGTTs were performed as previously described, using a 2 g/kg glucose load via oral gavage [8]. In preparation for the insulin tolerance test (ITT), fathers and offspring were fasted for 6 h at 11 and 15 weeks of age, respectively, and then given a 0.75 U/kg insulin load via intraperitoneal injection. Blood glucose measurements for both OGTT and ITTs were collected at baseline and 15, 30, 60, 90, and 120 min following the glucose or insulin load, using a One Touch Ultra^®^ 2 glucose meter (Lifespan, Burnaby, BC, Canada).

### 2.4. Tissue and Blood Collection

At 12 and 16 weeks of age, fathers and offspring respectively, underwent 12 h of food deprivation before euthanization. Animals were euthanized via over-anesthetization with isoflurane, followed by decapitation. Blood was collected from the portal vein in a chilled tube containing diprotinin-A (0.034 mg/mL blood; MP Biomedicals, Irvine, CA, USA), Sigma protease inhibitor (1 mg/mL blood; Sigma Aldrich, Oakville, ON, Canada), and Roche Pefabloc (1mg/mL of blood; Roche, Mississauga, ON, Canada). Samples were centrifuged and serum was collected and stored in −80 °C until insulin, peptide tyrosine tyrosine (PYY) and glucagon-like peptide 1 (GLP-1) analysis, using a Rat Metabolic Multiplex Array (Millipore, St. Charles, MO, USA) by Eve Technologies (Calgary, AB, Canada). To estimate insulin resistance, we used the formula for homeostatic model assessment of insulin resistance (HOMA-IR) = (glucose (mmol/L) × insulin (mIU/mL))/22.5 [14]. Heart, liver, kidney, cecum, colon, and testes (in males) were excised and weighed to determine organ weight, relative to body weight. A liver sample from the right lobe was collected and stored in in −80 °C until triglyceride assessment.

### 2.5. Triglyceride Concentration

Using 25 mg of liver, triglyceride concentrations were quantified in ug per mg of liver tissue using enzyme glycerol phosphate oxidase (GPO) reagents, according to manufacturer’s guidelines (Pointe Scientific Inc., Lincoln Park, MI, USA).

### 2.6. Gut Microbiota 16S rRNA Gene Sequencing

Paternal fecal matter was collected at baseline (3 weeks of age, prior to dietary interventions), 9 and 12 weeks of age. Offspring fecal matter was collected at weaning (3 weeks of age), 9 and 15 weeks of age. All fecal matter was stored at −80 °C until analysis. Bacterial DNA was extracted using the FastDNA spin kit for feces (MP Biomedicals, Lachine, QC, Canada) and brought to a concentration of 4 ng/uL. 16S rRNA gene sequencing of the V3 and V4 region took place at the Centre for Health Genomics and Informatics (University of Calgary, Calgary, AB, Canada) using the MiSeq Illumina platform (Illumina, San Diego, CA, USA) as previously described [12,13].

### 2.7. Cecal Short-Chain Fatty Acids (SCFAs)

SCFAs were measured in cecal matter collected at euthanasia from fathers and offspring as previously described [15]. Reverse-phase High Performance Liquid Chromatography (HPLC) using a c18 column containing a column guard was used to quantify the SCFA. An elution gradient of acetonitrile containing 0.05% trifluoracetic acid (8–100%) and a flow rate of 0.8 mL/min over 30 min was maintained.

### 2.8. Statistical Analysis and Taxonomy Profiling

Statistical analysis was conducted using IBM^®^ SPSS Statistics, version 24.0 except for 16S rRNA sequencing data. A multivariate, general linear model (GLM) was used to determine a sex effect between male and female offspring. If a sex effect was identified, males and females were analyzed separately using an independent samples *t*-test. Outcomes with multiple time points were analyzed using a repeated measures GLM, wherein diet was the “between-subject” factor and time was the “within-subject” factor. Identification of a significant interaction between diet and time was followed with an independent samples *t*-test to determine differences between dietary groups. All data was presented as mean ± standard error of the mean (SEM). Significance was set at *p* ≤ 0.05.

16S rRNA sequencing data was analyzed using the R statistical software as previously described [16]. Initially, data was quality filtered using the filterAndTrim, assignTaxonomy and assignSpecies functions using dada2 (version 1.10.1) [17]. The phyloseq package (version 1.24.2) [18] was used to determine diversity between groups. Alpha diversity was assessed using Chao1, Shannon and Simpson indices, where differences were analyzed using an independent *t*-test. Beta diversity was assessed using a principal coordinates analysis (PCoA) using a Bray–Curtis distance matrix, where a PERMANOVA was used to classify significance. LEfSe tool was used to determine differentially abundant features [19]. Significance was set at *p* ≤ 0.05.

## 3. Results

### 3.1. No Difference Was Observed in Bodyweights or Body Composition Intergenerationally

**Figure 1:**
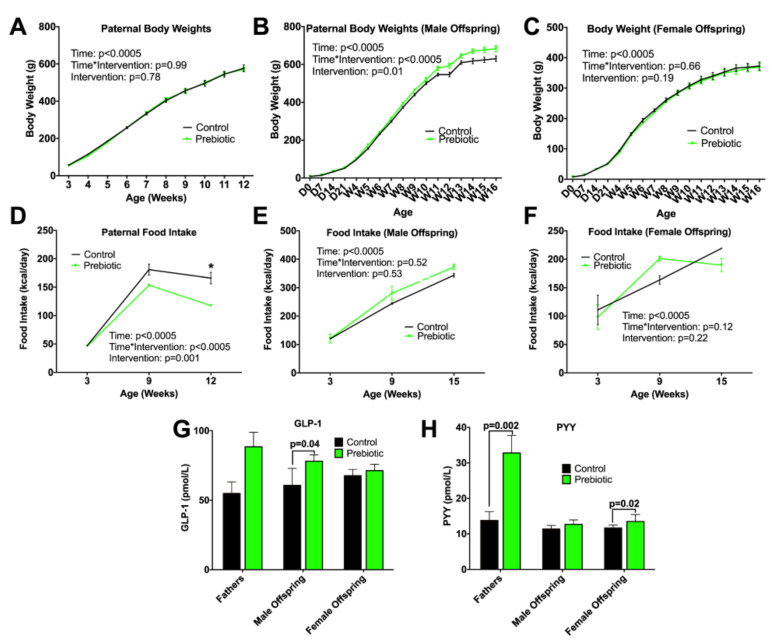
Body weight, food intake, and gastrointestinal hormones. Body weight of (**A**) fathers, (**B**) adult male offspring and (**C**) adult female offspring; food intake of (**D**) fathers, (**E**) male offspring (**F**) female offspring; (**G**) GLP-1 and (**H**) PYY. Food intake was analysed using independent samples Kruskal–Wallis tests. Values are means ± SEM, *n* = 8–13. In adult offspring, there was a significant sex effect in the overall model for bodyweight (*p* = 0.0001), food intake (*p* = 0.0001), GLP-1 (*p* = 0.002); therefore, subsequent analysis was performed in males and females separately. * represents a significant difference between groups, *p* < 0.05.

To assess whether there was a sex effect between male and female offspring, we conducted a multivariate GLM for all parameters. At every age and parameter (bodyweight, body composition, and organ weight), a sex difference was observed, therefore males and females were assessed separately. No difference between control and prebiotic was observed for body weight in fathers (Figure 1A), male (Figure 1B), or female (Figure 1C) offspring. Fathers showed significantly higher bone mineral content in the prebiotic group compared to control (*p* = 0.02), with a trend towards increased lean + BMC mass in fathers (*p* = 0.052) and adult male offspring (*p* = 0.08) (Table 2).

### 3.2. Larger Distal Gut Seen with Prebiotic Diet

Paternal cecum and colon mass were significantly higher in the prebiotic compared to control group (Table 3). Adult male offspring exhibited a trend (*p* = 0.08) towards increased testes weight in the prebiotic group (Table 3). Adult female prebiotic offspring showed significantly higher brain mass compared to control offspring (Table 3).

### 3.3. Gastrointestinal Hormones Were Increased in Fathers and Offspring

As expected, there was a significant main effect of time for food intake with intake increasing as the fathers, male offspring and female offspring aged (*p* < 0.0005; Figure 1). There was also a significant interaction between time and diet among fathers, with prebiotic fathers consuming significantly fewer kcal/day at 12 weeks of age (*p* < 0.0005; Figure 1D). No difference in food intake was observed in male or female offspring (Figure 1E,F).

We examined serum concentrations of glucagon-like peptide-1 (GLP-1) and peptide tyrosine tyrosine (PYY), two gastrointestinal hormones associated with satiety. GLP-1 was increased in adult prebiotic male offspring compared to controls (*p* = 0.04; Figure 1G). PYY was significantly increased in fathers consuming the prebiotic-rich diet (*p* = 0.002; Figure 1H). Higher PYY was similarly seen in adult female offspring from prebiotic-fed fathers (*p* = 0.02; Figure 1H).

### 3.4. Paternal Prebiotic Intake Did Not Affect Glucose or Insulin Homeostasis

**Figure 2:**
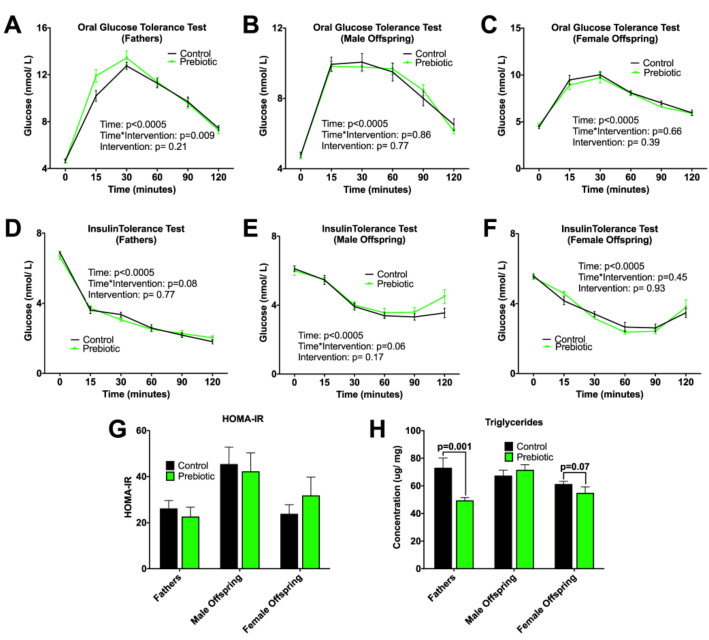
Oral glucose tolerance test (OGTT), insulin tolerance test (ITT), homeostatic model assessment of insulin resistance (HOMA-IR), and hepatic triglyceride concentrations. OGTT of (**A**) fathers, (**B**) adult male offspring and (**C**) adult female offspring; ITT of (**D**) fathers, (**E**) male offspring, (**F**) female offspring; (**G**) HOMA-IR; (**H**) triglyceride concentrations in hepatic tissue. Values are means ± SEM, *n* = 8–13. In adult offspring, there was a significant sex effect in the overall model for OGTT (*p* < 0.0001), ITT (*p* < 0.003), HOMA-IR (*p* = 0.04), triglyceride (*p* = 0.03), therefore subsequent analysis was performed in males and females separately, *p* < 0.05.

To determine whether paternal prebiotic intake affected metabolic outcomes intergenerationally, we conducted insulin and glucose tolerance tests. An OGTT measures the animal’s response to an oral glucose load, wherein, persistently elevated blood glucose levels reflect glucose intolerance [20]. During an ITT, lower glucose values over the 120 min test reflect greater insulin sensitivity [20]. There was a significant interaction between time and diet for glucose tolerance in fathers (Figure 2A). Due to the significant sex effect seen within both the OGTT and ITT (*p* < 0.0001), male and female adult offspring were assessed separately. No differences were observed in glycemia or insulin sensitivity in male and female offspring (Figure 2B,C,E,F). Similarly, no difference was seen in insulin resistance in fathers or adult offspring as determined by HOMA-IR (Figure 2G). We did, however, find intergenerational differences in hepatic triglyceride concentrations. Prebiotic fathers showed reduced hepatic triglyceride concentration compared to control (*p* = 0.001), which persisted in adult female offspring as a trend towards a decrease (*p* = 0.07) (Figure 2H).

### 3.5. Paternal Prebiotic Consumption Alters Gut Microbiota Signatures Intergenerationally

Using 16S rRNA gene amplicon sequencing, we revealed microbial community change intergenerationally. At three weeks of age, prior to dietary intervention, there were no differences in paternal alpha diversity (Table 4); however, at nine weeks of age onwards, prebiotic fathers showed a significant reduction in alpha diversity (*p* < 0.001, Table 4). Male prebiotic offspring at weaning showed reductions in alpha diversity as measured by the Chao1 index, however, this was not observed using Shannon or Simpson indices (Table 4). Female prebiotic offspring showed significantly higher alpha diversity compared to controls, as measured by Chao1, Shannon and Simpson indices at weaning. This increased alpha diversity persisted until nine weeks of age (*p* < 0.002, Table 4).

**Figure 3:**
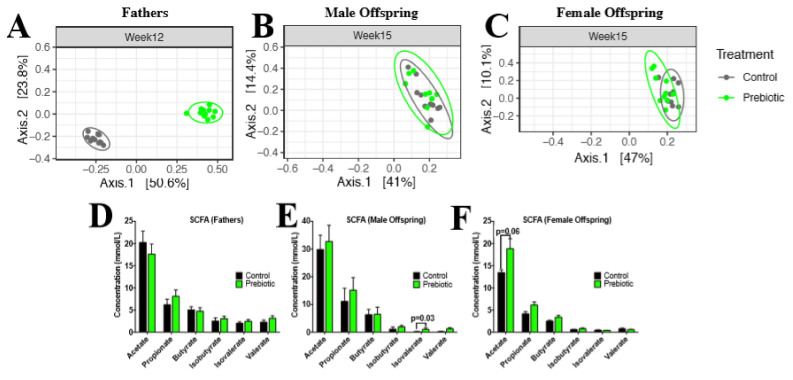
Fecal microbiota comparisons of fathers fed prebiotic or control diet and the intergenerational similarities in male and female offspring. Beta diversity of (**A**) paternal at 12 weeks of age, (**B**) male offspring and (**C**) female offspring at 15 weeks of age, calculated with principal coordinates analysis (PCoA) using a Bray–Curtis distance matrix. Cecal short chain fatty acids in: (**D**) fathers, (**E**) adult male offspring and (**F**) adult female offspring at euthanasia. Values are means ± SEM, *n* = 8–13 (*p* < 0.05).

To assess bacterial clustering based on paternal diet, we performed a principal coordinates analysis (PCoA), wherein points that are closer together signify microbial communities that have sequence compositions that are more alike, whereas microbial communities that are further apart and significant denote microbial sequence compositions that more disparate. As expected, fathers showed significant differences in beta diversity between dietary groups at 9 and 12 weeks of age (*p* < 0.001, Figure 3A). Female offspring also showed significant differences in beta diversity at 15 weeks of age, showing larger dispersion in the prebiotic group compared to controls (*p* = 0.04, Figure 3C). Male offspring did not exhibit any differences in beta diversity at any age (Figure 3B). To further elucidate the microbial differences between dietary interventions, we used a linear discriminant analysis (LDA) effect size (LEfSe) tool, which reveals significant differences in bacterial abundances between control and prebiotic fathers or offspring.

**Figure 4:**
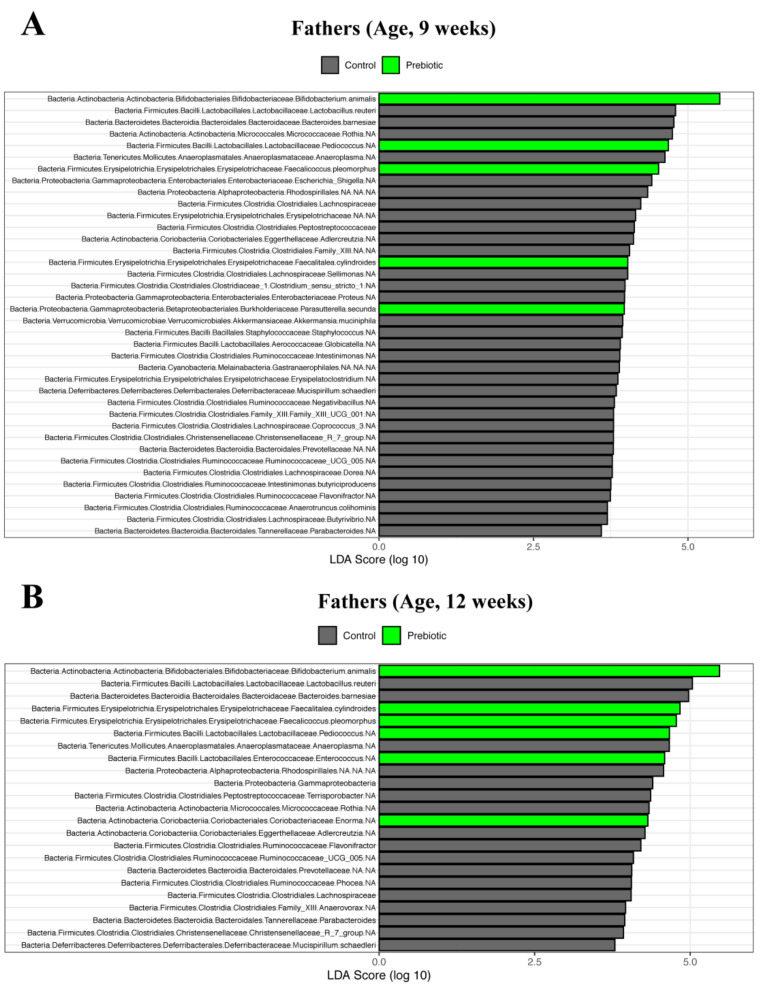
Fecal microbiota comparisons using a LEfSe tool of fathers fed prebiotic or control diet. (**A**) fathers at 9 weeks of age, (**B**) fathers at 12 weeks of age.

At the phylum level, Actinobacteria were the only significantly increased bacteria in fathers at 9 (Figure 4A) and 12 (Figure 4B) weeks of age. This increase was owing to the increased abundance of the *Bifidobacterium* genera at 9 and 12 weeks of age, showing a notable increase in *Bifidobacterium animalis* (Figure 4A,B). Furthermore, Lactobacillaceae and Erysipelotrichaceae were increased, with two prominent genera within each family of bacteria—*Pediococcus* and *Streptococcus* in the Lactobacillaceae family and *Faecalicoccus* and *Faecalitalea*—belonging to the Erysipelotrichaceae family in prebiotic fathers (Figure 4A,B). At 12 weeks of age, *Enorma* was also significantly increased in prebiotic fathers (Figure 4B).

**Figure 5:**
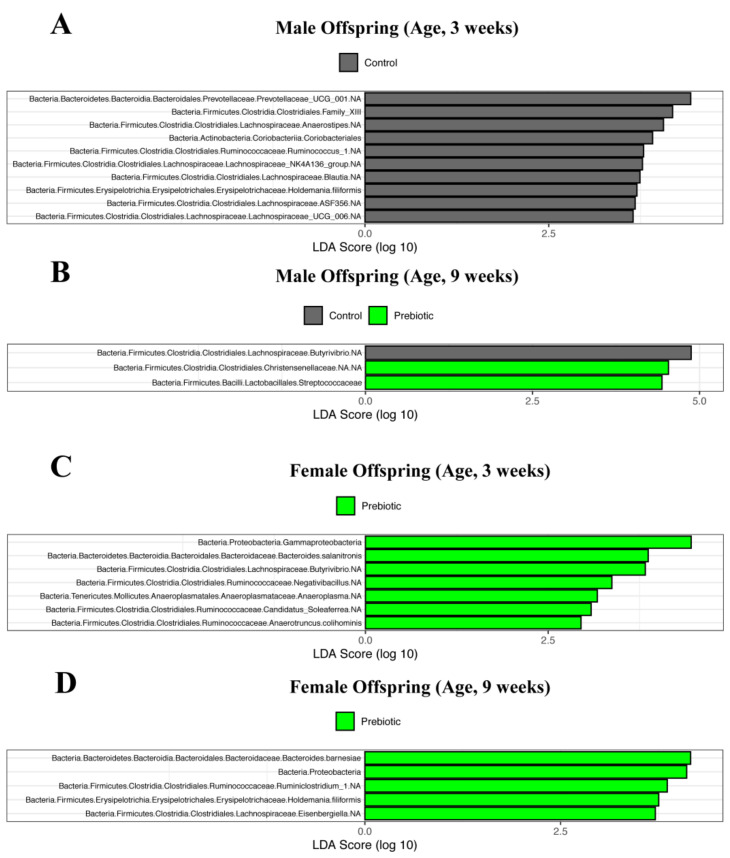
Fecal microbiota comparisons using a LEfSe tool of male and female offspring sired from fathers fed prebiotic or control diets for 9 weeks. (**A**) male offspring at 3 weeks of age, (**B**) male offspring at 9 weeks of age, (**C**) female offspring at 3 weeks of age, (**D**) female offspring at 9 weeks of age.

Male prebiotic offspring at nine weeks of age showed significantly increased Christensenellaceae and Streptococcaceae (Figure 5B). Female prebiotic offspring at weaning showed an increase in the Tenericutes phylum driven almost entirely by the genus *Anaeroplasma* (Figure 5C). Female prebiotic offspring showed significant increases in Bacteroidetes phylum at nine weeks of age (Figure 5D). No differences were seen in male or female offspring at 15 weeks of age.

### 3.6. Paternal Prebiotic Intake Affects Offspring SCFA in Cecal Matter

Since prebiotics are indigestible by the host, they reach the colon, largely intact, serving as carbon sources for bacterial fermentation, thereby yielding SCFAs as end products [21,22]. No differences were seen in cecal concentrations of acetate, propionate, butyrate, isobutyrate, isovalerate, and valerate between dietary groups in fathers at 12 weeks of age (Figure 3A). Male prebiotic offspring showed a significant increase in isovalerate at 16 weeks of age (Figure 3B). Female prebiotic offspring showed a trend (*p* = 0.06) towards increased acetate at 16 weeks of age (Figure 3C).

## 4. Discussion

Although clear beneficial metabolic effects have been observed for both mother and offspring with maternal prebiotic intake during gestation and lactation [5,6,7,8,23], our findings suggest that paternal prebiotic intake before conception, improves metabolic and gut microbial status in fathers but has only limited impact on offspring health. The effects seemed to be largely confined to increased serum PYY, a trend towards decreased hepatic triglyceride concentrations and increased cecal acetate in female offspring and only increased serum GLP-1in male offspring. Minimal gut microbiota alterations were seen in male and female prebiotic offspring compared to control offspring.

We did not observe any differences in bodyweight or adiposity in fathers consuming oligofructose, which is not entirely consistent with previous work in rodents although it is important to note that the majority of studies showing reduced body fat involve the addition of a prebiotic to an obesogenic high fat/high sucrose diets rather than a control diet such as we did [13]. It is possible therefore, that the metabolic impact of paternal oligofructose consumption could be more evident in an obese paternal model where fathers are consuming a high fat/high sucrose diet although this remains to be examined.

Despite the lack of effect of paternal oligofructose intake on body weight or body composition, we did see a notable decrease in hepatic triglyceride concentrations in fathers and female offspring. The neutral storage form of fatty acids is triglycerides whose metabolism occurs primarily in hepatocytes [24]. The liver is the primary organ that modulates lipid homeostasis using complex biochemical, signaling, and cellular mechanisms [24]. In a healthy subject, the liver processes vast amounts of fatty acids and only stores a small amount as triglycerides [25]. Excess triglyceride accumulation is typical of diseased states that affect the liver, including type 2 diabetes, dyslipidemia, as well as increased incidence of insulin resistance [26]. Nevertheless, we did not observe any differences in measures of insulin resistance, as determined by HOMA-IR, OGTT, or ITT, which may not be surprising given that the rats were all fed a control diet and this was not an obese, insulin resistant model which might make differences more apparent between groups.

Our paternal microbial results reflect that of multiple animal and human studies over the past few decades, showing that prebiotic intake promotes the proliferation of *Bifidobacterium* [8,12,27,28]. Specifically, we saw an increased abundance of *B. animalis*, a species previously linked to reductions in or slowing down the accumulation of adiposity [29] and low-grade, chronic, and systemic inflammation [30]. Prebiotic consumption is also typically accompanied by the proliferation of *Lactobacillus* [31], which was similarly consistent with our findings in fathers. This is a biological advantage, since *Lactobacillus* microbes, like *L. plantarum* [32] and *L. reuteri* [33], are natural producers of B vitamins, including B1–3, B6, B8, B9, and B12, all of which play a vital role in immune regulation and metabolic health.

Prebiotics, and particularly chicory root-derived oligofructose and inulin have been shown to increase the L cell numbers in the distal gut which are responsible for the production of the satiety hormones GLP-1 and PYY [34,35,36]. This fits with the increased serum PYY and reduced energy intake we observed in fathers consuming oligofructose in our study. Interestingly, male offspring of prebiotic fathers had increased GLP-1 while female offspring had increased PYY, although this did not translate into reduced energy intake in either sex. We postulate that if we followed male and female offspring, sired from prebiotic or control fathers, beyond four months of age, we may have observed more pronounced metabolic differences. This might have been especially true if the offspring were subjected to a high fat/high sucrose diet challenge which may have unmasked latent metabolic effects, including increased energy intake or metabolic disease risk, which OFS may have been able to mitigate via altered satiety hormone levels.

Given that prebiotics are known to exert a substantial effect on gut microbiota [35], it is not surprising that the fathers who directly consumed the oligofructose would have substantial shifts in their gut microbiota compared to controls. What is more intriguing is that we also observed some although limited microbiota compositional shifts in their offspring. First, female offspring showed significantly increased alpha diversity compared to controls at weaning and nine weeks of age. Female prebiotic offspring at weaning showed a significant increase in *Anaeroplasma*. *Anaeroplasma* belongs to the Tenericutes phylum, which has been associated with beneficial effects on gastrointestinal (GI) health, modulating intestinal integrity [37]. In instances of GI inflammation in previous work, Tenericutes were substantially reduced [37]. Moreover, a reduced abundance of *Anaeroplasma* spp. has been associated with fecal hardness and gut microbial dysbiosis [38].

Female offspring at nine weeks of age showed enriched microbial composition of Bacteroidetes. Sonnenburg and colleagues [39] showed significantly increased abundance of species belonging to the Bacteroidetes phylum, specifically *Bacteroides* spp., which proliferated in response to prebiotic fructans. Specifically, *Bacteroides caccae*, *Bacteroides fragilis*, *Bacteroides ovatus*, *Bacteroides uniformis*, and *Bacteroides vulgatus* all increased [39]. Importantly, as previously mentioned, bacterial species feed on non-digestible dietary fibers, like oligofructose, producing metabolites like SCFA [40]. SCFA confer beneficial effects on the intestinal mucosa [40]. Members of the Bacteroidetes phylum primarily produce acetate and propionate [41]. Female offspring in our study, although they did not directly consume the oligofructose, showed increased abundance of the Bacteroidetes phylum as well as a concurrent increase in cecal acetate levels. Acetate plays a role in cholesterol metabolism and lipogenesis as well as satiety regulation, and has recently been shown to play a role in the browning of white adipose tissue [42,43]. One study in a rabbit model showed that acetate decreased hepatic triglyceride concentration by inhibiting fatty acid synthesis and promoting fatty acid oxidation [44]. They also found beneficial effects of acetate on skeletal muscle and adipose tissue triglyceride levels and fat content [44]. This may provide an explanation of the reduction in triglyceride content in liver in female offspring who also exhibited increased acetate levels. Moreover, a large correlation cohort, involving 893 subjects, previously showed that fecal alpha diversity was negatively correlated with blood triglycerides [45], which may further explain the pattern (*p* = 0.07) of decreased hepatic triglycerides we saw in female offspring.

The effects of paternal prebiotic intake on male offspring gut microbiota were limited but did include an increase in the abundance of Christensenellaceae at nine weeks of age. Christensenellaceae has been associated with lean body mass, longevity, and the absence of metabolic syndrome [46]. Interestingly, Christensenellaceae has been shown to be highly heritable in many different populations [46].

Multiple studies have assessed the impact of maternal prebiotic intake on offspring health [7,8]. This study expands the parental impact of prebiotic diets to also include father’s intake of oligofructose before conception. The effects of the direct consumption of prebiotics by fathers are consistent with the known bifidogenic and satiety-promoting effects of oligofructose. In offspring there were relatively few metabolic changes, but female offspring were impacted to a greater extent than males. Future work is warranted to assess the sex-dependent intergenerational transmission of microbial and metabolic impacts of parental diets. Presently, it remains unclear how fathers may affect offspring gut microbial or metabolic outcomes, since paternal microbes cannot be vertically transmitted to offspring similar to how mothers contribute to the colonization of the infant gut at birth. Although, no one has addressed this, we speculate that by improving offspring metabolic health due to paternal dietary patterns, there will be a concurrent improvement in gut microbial signatures in offspring. To causally link offspring gut microbiota to metabolic outcomes, fecal microbiota transplant studies utilizing a gnotobiotic mouse model is warranted.

## Figures and Tables

**Table 1:** Experimental diet composition from 3 to 9 and 10 to 16 weeks of age.

	Control	Prebiotic	Control	Prebiotic
Grams	Weeks 3–9		Weeks 10–16	
Cornstarch	397.5	357.8	465.7	419.13
Casein	200	180	140	126.0
Dyetrose	132	118.8	155	139.5
Sucrose	100	90	100	90
Soybean Oil	70	63	40	36
Alphacel	50	45	50	45
AIN-93M Mineral Mix	35	31.5	35	31.5
AIN-93 Vitamin Mix	10	9	10	9
L-cystine	3	2.7	1.8	1.62
Choline-Bitartrate	2.5	2.25	2.5	2.25
Oligofructose	0	100	0	100
Energy density (kJ/g)	15.7	14.8	15.1	14.2
Carbohydrate (% of kcal)	63.9	65.4	75.9	76.9
Protein (% of kcal)	19.4	18.6	14.1	13.5
Fat (% of kcal)	16.8	16.0	10.0	9.6
Total fiber (g)	50	145	50	145

Control diets were AIN-93G and AIN-93M respectively for weeks 3–9 and weeks 10–16. Diets were purchased from Dyets, Inc. (Bethlehem, PA, USA). Alphacel is an insoluble fiber of powdered cellulose. Prebiotic diets were mixed in-house by combining 900 g of control diet with 100 g of oligofructose (Orafti P95, Beneo, Mannheim, Germany).

**Table 2:** Body composition of fathers at 12 weeks of age and offspring at 16 weeks of age ^1^.

	Control	Prebiotic	*p*-Value
Fathers			
BMC (g)	15.0 ± 0.3	15.6 ± 0.2	0.02
BMD (g/cm^2^)	0.166 ± 0.001	0.167 ± 0.002	0.44
Fat mass (g)	85.5 ± 5.8	87.3 ± 6.9	0.57
Lean + BMC (g)	504.1 ± 14.6	516.5 ± 8.7	0.05
Body fat (%)	15.0 ± 0.9	14.3 ± 0.9	0.97
Males			
BMC (g)	17.2 ± 0.4	17.4 ± 0.3	0.28
BMD (g/cm^2^)	0.175 ± 0.002	0.172 ± 0.001	0.68
Fat mass (g)	108.2 ± 7.0	144.4 ± 11.2	0.11
Lean + BMC (g)	519.5 ± 12.0	521.4 ± 6.3	0.08
Body fat (%)	19.5 ± 1.6	21.0 ± 1.4	0.60
Females			
BMC (g)	11.6 ± 0.3	11.3 ± 0.3	0.70
BMD (g/cm^2^)	0.168 ± 0.002	0.166 ± 0.002	0.40
Fat mass (g)	78.0 ± 8.1	76.9 ± 9.8	0.74
Lean + BMC (g)	287.5 ± 7.7	286.3 ± 10.0	0.49
Body fat (%)	21.1 ± 1.7	20.5 ± 2.2	0.96

^1^ Values are mean ± SEM, *n* = 9–13/group. BMC, bone mineral content; BMD, bone mineral density. Male BMC was log transformed for analysis.

**Table 3:** Organ weight of fathers at 12 weeks of age and offspring at 16 weeks of age ^1^.

	Control	Prebiotic	*p*-Value
Fathers			
Body weight (g)	594.2 ± 19.5	611.3 ± 12.3	0.09
Organ weight (%BW)			
Heart	0.32 ± 0.01	0.28 ± 0.01	0.43
Liver	3.04 ± 0.07	2.86 ± 0.06	0.31
Kidney	0.27 ± 0.01	0.28 ± 0.004	0.11
Cecum	0.10 ± 0.004	0.29 ± 0.01	0.03
Colon	0.21 ± 0.01	0.23 ± 0.01	0.002
Testes	0.31 ± 0.01	0.31 ± 0.01	0.15
Males			
Body weight (g)	630.5 ± 14.4	683.6 ± 15.9	0.02
Organ weight (%BW)			
Heart	0.27 ± 0.01	0.26 ± 0.01	0.40
Liver	2.69 ± 0.01	2.72 ± 0.01	0.87
Kidney	0.26 ± 0.01	0.25 ± 0.01	0.29
Cecum	0.09 ± 0.002	0.10 ± 0.003	0.82
Colon	0.27 ± 0.01	0.30 ± 0.01	0.10
Testes	0.16 ± 0.01	0.17 ± 0.01	0.08
Brain	0.35 ± 0.01	0.33 ± 0.01	0.60
Females			
Body weight (g)	372.6 ± 13.5	368.8 ± 12.2	0.20
Organ weight (%BW)			
Heart	0.32 ± 0.01	0.20
Liver	2.65 ± 0.05	2.97 ± 0.06	0.85
Kidney	0.27 ± 0.01	0.28 ± 0.01	0.71
Cecum	0.12 ± 0.01	0.13 ± 0.01	0.48
Colon	0.25 ± 0.01	0.28 ± 0.01	0.14
Brain	0.55 ± 0.03	0.57 ± 0.02	0.02

^1^ Values are mean ± SEM, *n* = 9–13/group. Paternal colon weight and female kidney weight was log transformed for analysis.

**Table 4:** Alpha diversity at three different age groups in fathers and offspring ^1^.

	Control	Prebiotic	*p*-Value
3 Weeks of Age			
Fathers			
Chao1	294.05 ± 24.13	282.82 ± 27.96	0.75
Shannon	3.93 ± 0.08	3.85 ± 0.11	0.65
Simpson	0.94 ± 0.01	0.94 ± 0.01	0.52
Male Offspring			
Chao1	181.01 ± 21.85	111.38 ± 15.29	0.02
Shannon	3.97 ± 0.07	3.77 ± 0.08	0.06
Simpson	0.96 ± 0.003	0.96 ± 0.003	0.68
Female Offspring			
Chao1	210.13 ± 37.54	335.67 ± 30.16	0.002
Shannon	3.94 ± 0.09	4.22 ± 0.06	0.0005
Simpson	0.96 ± 0.003	0.96 ± 0.002	0.022
9 Weeks of Age			
Fathers			
Chao 1	225.34 ± 14.2	44.64 ± 3.51	0.001
Shannon	3.75 ± 0.08	2.27 ± 0.08	0.001
Simpson	0.94 ± 0.004	0.82 ± 0.01	0.001
Male Offspring			
Chao 1	120.01 ± 24.76	138.22 ± 16.55	0.18
Shannon	3.42 ± 0.13	3.62 ± 0.06	0.069
Simpson	0.93 ± 0.01	0.94 ± 0.003	0.070
Female Offspring			
Chao 1	124.93 ± 11.43	166.25 ± 15.58	0.002
Shannon	3.45 ± 0.08	3.72 ± 0.08	<0.0001
Simpson	0.93 ± 0.006	0.95 ± 0.004	<0.0001
12 and 15 Weeks of Age			
Fathers			
Chao 1	149.09 ± 10.88	39.36 ± 2.23	<0.0001
Shannon	3.76 ± 0.06	2.51 ± 0.04	<0.0001
Simpson	0.95 ± 0.002	0.87 ± 0.01	<0.0001
Male Offspring			
Chao 1	109.62 ± 7.41	107.44 ± 15.85	0.10
Shannon	3.45 ± 0.10	3.58 ± 0.17	0.059
Simpson	0.93 ± 0.01	0.94 ± 0.01	0.13
Female Offspring			
Chao 1	156.80 ± 19.58	168.50 ± 29.49	0.85
Shannon	3.65 ± 0.13	3.76 ± 0.13	0.78
Simpson	0.94 ± 0.01	0.95 ± 0.01	0.79

^1^ Values are mean ± SEM, *n* = 9–11/group.

## Data Availability

Data is available from the corresponding authors upon reasonable request.
